# Integrative network pharmacology, molecular docking, and dynamic simulation analysis of a polyherbal formulation for potential therapeutic impact on prostate cancer

**DOI:** 10.1016/j.heliyon.2024.e34531

**Published:** 2024-07-11

**Authors:** Ansari Vikhar Danish Ahmad, Syed Ayaz Ali, Qazi Yasar, Nikhil S. Sakle, Mohd Mukhtar Khan

**Affiliations:** Dr. Rafiq Zakaria Campus, Y. B. Chavan College of Pharmacy, Aurangabad, 431001, Maharashtra, India

**Keywords:** Network pharmacology, Molecular docking, Dynamic simulations, Prostate Cancer, Polyherbal formulation

## Abstract

**Background:**

Prostate cancer (PCa) remains a significant health concern globally, prompting a continual search for novel therapeutic strategies. In this study, we employed a comprehensive approach combining network pharmacology, molecular docking and dynamic simulation to explore the potential impact of a polyherbal formulation on PCa.

**Methods:**

Utilizing comprehensive network pharmacology approaches, we elucidated the complex interactions between the bioactive compounds within the polyherbal formulation and key targets associated with PCa progression, highlighting their multitarget mechanisms through integrated protein‒protein interaction and KEGG pathway analyses. Molecular docking simulation studies were performed to predict the binding affinities and modes of interaction between the identified bioactive compounds and their respective protein targets.

**Results:**

Complex connections comprising 486 nodes and 845 edges were found by the compound-target network analysis. Significant interactions were observed, and the average node degree was 4.23. KEGG research revealed that PCa and the PI3K-Akt signalling pathway are implicated in modulating prostate cancer. The Quercetin docking investigations revealed that the binding energies for AR and PIK3R1 were −9 and −9.5 kcal/mol, respectively. Based on the results of the MD simulations, it appears that tiny molecules and proteins have formed stable complexes with low fluctuations.

**Conclusion:**

In conclusion, this comprehensive method emphasises the value of network pharmacology in conjunction with molecular docking and dynamic simulation in revealing the anti-PCa therapeutic potential of polyherbal formulations, opening up new possibilities for the creation of efficient anti-cancer medicines.

## 1 Introduction

Prostate cancer, a malignant tumor, poses a significant threat to life, ranking highest in terms of incidence and second highest in mortality among males [1,2]. Symptoms of prostate cancer are often unclear, and it typically advances to later stages before obvious symptoms manifest [3]. Advanced prostate cancer, notably castration-resistant prostate cancer (CRPC), exhibits heightened metastasis, aggressiveness, and resistance to treatment, often progressing to the terminal stage [4,5]. Androgen is an important factor in prostate cancer progression [6]. with endocrine therapy serving as a vital treatment modality for advanced cases [7]. Black cumin, or Nigella sativa, has a long history in traditional medicine because of its many health advantages. Its primary compound, thymoquinone and quercetin, acts as a potent antioxidant and anti-inflammatory substance [[8], [9], [10]]. Studies suggest that Black Seed (*Nigella sativa*) may have potential anticancer effects by inhibiting the growth of cancer cells and triggering programmed cell death, known as apoptosis [11]. Furthermore, research has explored its therapeutic potential in managing conditions such as diabetes, asthma, and cardiovascular diseases [12]. Garlic (*Allium sativum*), a fundamental ingredient in many culinary dishes, has been esteemed for its medicinal properties for thousands of years. Allicin, the sulfur compound responsible for garlic's distinct smell and taste, is being investigated for its potential anticancer properties. Research indicates that allicin can impede the proliferation of cancer cells, thereby reducing the risk of certain cancers like stomach and colorectal cancer [13,14]. Garlic is also known for its antibacterial, antiviral, and immune-boosting properties, as well as its ability to regulate cholesterol levels [15]. Honey (*Apis mellifera*), a natural sweetener with a rich medicinal history, contains a wealth of antioxidants and phytochemicals. Its antibacterial, antiviral, and anti-inflammatory properties make it valuable in addressing various ailments, including cancer. Some studies suggest that honey may have anticancer properties by inhibiting the growth of cancer cells [16]. Additionally, honey's traditional use in wound healing, as well as its effectiveness in soothing sore throats and coughs, further underscore its therapeutic value [17].

Traditional medicines exhibit pharmacological characteristics targeting multiple pathways. The content of the polyherbal formulation has vast biological properties [Table S1]. To identify potential targets of a Polyherbal formulation for prostate cancer treatment, we employed network pharmacology. Initially, this involved analyzing network communication predictions and formulation targets. By employing network pharmacology, we were able to pinpoint the active ingredients and therapeutic targets for prostate cancer treatment. Afterwards, these targets were predicted using gene ontology and KEGG pathway enrichment studies. Notably, the PI3K/Akt signaling pathway, crucial for vascular endothelial cell apoptosis, was investigated due to its role in abnormal vascular network formation. This study delved into the impact of the Polyherbal formulation on prostate cancer, shedding light on its potential therapeutic effects. In this research, we seek to investigate the potential impact of a Polyherbal formulation on prostate cancer through both computational modeling and laboratory experiments. We employed predictive modeling to identify the active compounds' targets and constructed a network to illustrate the interactions between drugs and targets. Furthermore, we utilized molecular docking and dynamic simulations to verify these targets, evaluating the strength and stability of the interactions between compounds and targets. By unraveling the molecular mechanisms underlying the Polyherbal formulation's activity against prostate cancer, we aim to contribute to the development of novel therapies and targeted interventions for this serious ailment.

## 2 Materials and methods

### 2.1 Network pharmacology-based analysis screening of active components and construction of the interaction network in polyherbal formulation

The Polyherbal formulation's active ingredients and target genes were identified using the imppat database (https://cb.imsc.res.in/imppat/). Then, according to certain criteria, such as an oral bioavailability (OB) of 30 % or higher and a drug likeness (DL) of 0.18 or higher, the bioactive components of the Polyherbal formulation were identified. The screening was based on these conditions. In order to generate the targets for the active components, Cytoscape 3.7.1 Software (https://cytoscape.org/) was used. The picture depicts the association between the active ingredients of the Polyherbal formulation and the targets associated with prostate cancer. Each “node" represents each active ingredient and its target, while each “edge" shows the same relationship.

### 2.2 Screening of disease targets for prostate cancer

To investigate the potential targets related to “prostate cancer," we used the Disgenet database (https://www.disgenet.org/).

### 2.3 Venn diagram and PPI network construction

We used the Venn tool, which can be found at (https://bioinformatics.psb.ugent.be/webtools/Venn/), to better understand the connections between prostate cancer targets and the possible targets of a Polyherbal formulation. A Protein-Protein Interaction (PPI) network, which is a shared target interaction network, was built using STRING (https://cn.string-db.org/). The proteins were classified as “Homo sapiens" and the confidence level was set to “highest confidence: 0.9."

### 2.4 Gene ontology (GO) analysis and KEGG pathway enrichment

Annotation of pathways in the Gene Ontology (GO) and the Kyoto Encyclopedia of Genes and Genomes (KEGG) was carried out using the Enrichr and ShinyGo platforms, respectively, as described in [18]. In order to improve the accuracy of data prediction, the GO analysis was carried out to examine the gene cluster within the network. Incorporating carefully selected and predicted gene annotations resulting from these terms across different species, GO provides a methodically organised collection of many standardised terms pertaining to biological processes, molecular activities, and cellular components. An important tool for pathway enrichment analysis is the GO annotation of biological processes; this allows researchers to zero in on the most important biological processes for outlining relevant functional information relevant to the study's aims. We also used KEGG to look at the metabolic pathways and gene functions in our network of chemicals and genes. Its value is in the light it sheds on the complex relationship between molecular entities and disease mechanisms by revealing the pathways that contribute to the illness phenotype.

### 2.5 Molecular docking

#### 2.5.1 Software tools

Molecular docking studies made use of numerous tools and resources, including PyRx-Virtual Screening Tool, Autodock Vina, Pymol, Discovery Studio Visualizer 2020, UCSF ChimeraX, PDB, and PubChem. This method allowed for a comprehensive and varied analysis of the selected ligands' molecular interactions and binding strengths with the target proteins.

#### 2.5.2 Ligand preparation

From the official website of the U.S. National Library of Medicine, PubChem (https://pubchem.ncbi.nlm.nih.gov/), we received the ligands and an approved medicine (doxorubicin). Energy minimization (optimisation) followed the import of these structures into PyRx 0.8 with the open babel tool. Utilizing the Universal Force Field, this optimisation technique took into account essential aspects like the element, hybridization, and connection [19]. Later on, the ligand was changed to PDBQT format, which is the AutoDock Ligand standard.

#### 2.5.3 Target preparation

For the investigation, the researchers used the 3D models of PIK3R1 (PDB: 4JPS) and AR (PDB: 5JJM). The RCSB has made available for download the crystal structures of 4JPS (https://www.rcsb.org/structure/4JPS) and 5JJM (https://www.rcsb.org/structure/5JJM). Chimera at UC San Francisco visualised the structure that was downloaded. Using Discovery Studio Visualizer 2020, we cleaned up the target structures, removed any excess water molecules and bound ligands from the protein structures. Then, we saved the structures back to the original folder in PDB format, ready for docking. A docking study of doxorubicin and certain chosen ligands was conducted using AutoDock Vina 1.1.2 in PyRx 0.8 [20].

#### 2.5.4 Docking procedure

The docking software PyRx0.8 was used to load the purified target structures. The load molecule option was located on the File tool bar. After that, we used the right-click menu to change the receptor structure to AutoDock macromolecule (PDBQT format). The Vina Wizard Tool in PyRx 0.8 was used to conduct binding affinity tests. For the docking process, molecules (PDBQT files) including ligands and targets were chosen. The three-dimensional grid box 4JPS5JJM (size_x = 166.443789903 A^o^ , size_y = 109.716576851 A^o^ , size_z = 128.117889016 A^o^ ) for PIK3R1 (PDB: 4JPS), (size_x = 102.365594406 A^o^ , size_y = 77.4209958649 A^o^ , size_z = 93.4266535187 A^o^) for AR (PDB: 5JJM) was created using the AutoDock tool 1.5.6 with an exhaustiveness value of 8. This was done for the purpose of molecular docking simulation. To define the cavities, we first chose the molecules, and then we used PyRx's toggle selection spheres option to pick the active amino acid residues. The grid box was positioned correctly to occupy all the active binding sites and important residues. The ligands and targets were then docked to find out how well they got along after this alignment.

#### 2.5.5 Identification of cavity and active amino acid residues

The BIOVIA Discovery Studio Visualizer (version-19.1.0.18287) was used to identify the active amino acid residues in the protein. To analyse all the docking poses, ligand and protein contacts, and to determine the kinds of interactions (e.g., H-bonding, hydrophobic, and 2D interactions), the output files were imported into BIOVIA Discovery Studio Visualizer and UCSF Chimera.

#### 2.5.6 Molecular dynamic simulation

Molecular dynamics (MD) simulations lasting 100 nanoseconds (ns) were carried out using Desmond, a programme created by Schrodinger LLC. The chosen compounds' binding assessments to the target protein were computed using the receptor-ligand docking methodology within the realm of molecular dynamics simulation. Incorporating Newton's classical equation of motion, MD simulation analysis was performed to predict the ligand binding status in a physiological environment. With the help of Maestro's Protein Preparation Wizard, the chosen ligands and proteins went through minimization and optimisation processes. Discordant geometries, unwanted contacts, and steric conflicts were corrected. An orthorhombic box-featured solvent model called TIP3P (Intermolecular Interaction Potential 3 Points Transferable) was used in conjunction with the OPLS 2005 force field to generate the systems using the System Builder tool. The models were neutralised by adding counter ions, and physiological circumstances were mimicked by adding 0.15 M sodium chloride, all while keeping the temperature at 300 K and the pressure at 1 atm throughout the simulation. For further review, trajectories were saved at 100 ps intervals, and the stability of the protein-ligand combination was confirmed by time-dependent Root Mean Square Deviation (RMSD) analysis [21].

#### 2.5.7 Prime MM-GBSA analysis

In order to determine the binding free energy (Gbind) of the docked complex between Quercetin-4JPS and Quercetin-5JJM during MD simulations, the MM-GBSA tool inside Prime was used. One way to calculate the binding free energy is by using the VSGB solvent model, rotamer exploration methods, and the OPLS 2005 force field. After the MD simulation, frames for the MD trajectory were chosen at 10-ns intervals. Equation (1) was used to obtain the overall binding free energy:

1 dGbind = Gcomplex – (Gprotein + Gligand)------------1

The binding free energy is denoted as dGbind, the complex free energy as Gcomplex, the target protein free energy as Gprotein, and the ligand free energy as Gligand.

## 3 Results

### 3.1 Construction of active ingredient target interaction network

**Fig. 1:**
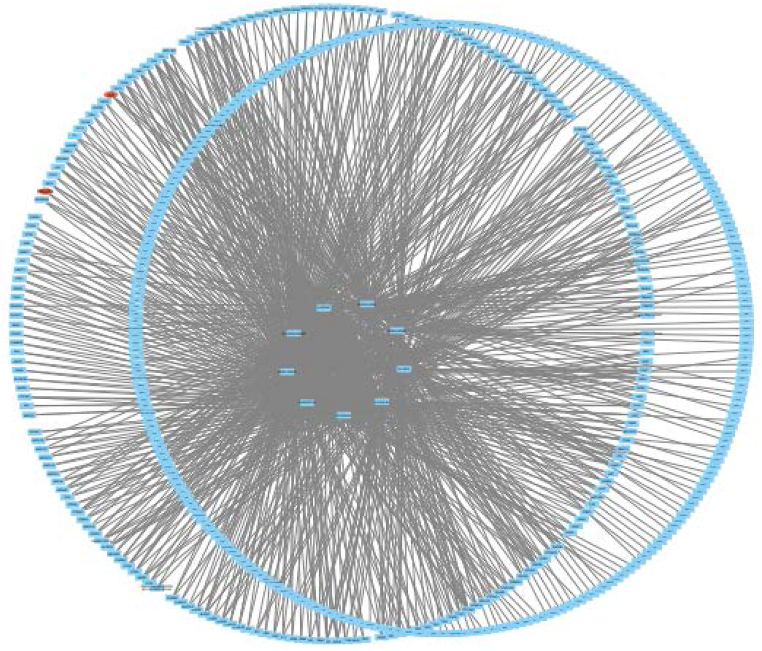
Compound-target-prostate cancer network constructed by Cytoscape v_3.7.1.

Using the TCMSP database, we screened for active ingredients in a Polyherbal formulation that had an oral bioavailability (OB) of 30 % or higher and a drug likeness (DL) score of 0.18 or higher. The nine bioactive components, including quercetin, thymoquinone, and catechin, were identified using the Cytoscape 3.7.1 programme. After that, an interaction network was constructed by compiling 845 distinct targets linked to these active components. The bioactive elements took the stage in this network, with blue nodes representing possible targets. The number of connections between nodes in a network was referred to as its degree of connectedness. The pharmacological efficacy of the Polyherbal formulation was determined to be heavily dependent on active components with a high degree of value (Fig. 1).

### 3.2 Construction and analysis of the target PPI network

**Fig. 2:**
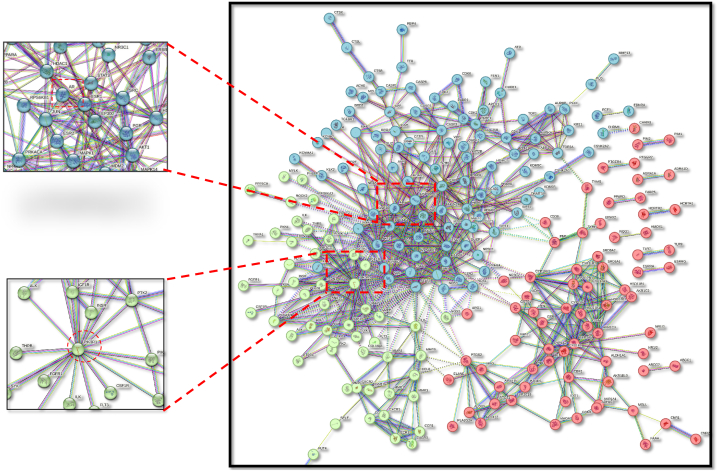
Protein–protein interaction network of Polyherbal formulation in prostate cancer obtained from STRING v_11.0 database.

An in-depth analysis of the target genes linked to each component was carried out using STRING v_11 to build and visualise the PPI network. A rigorous criterion was established at a score level higher than 0.9 to filter the high-confidence data on target protein interactions. Fig. 2 shows the final PPI network, which included 291 nodes and 616 edges, with each edge representing a different PPI. Importantly, 4.23 was found to be the average node degree, which represents the number of linked targets within the network. The quantity of targets linked to the network is correlated with a local clustering coefficient of 0.462. Key targets involved in the control of prostate cancer were identified by the study of the PPI network. These targets include PIK3R1, SRC, STAT3, HSP90AA1, AKT1, MAPK1, ESR1, and AR.

### 3.3 GO gene enrichment analysis and KEGG pathway annotation

**Fig. 3:**
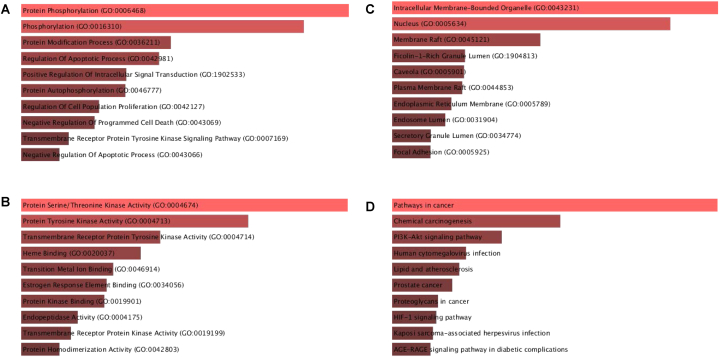
A) Biological Process, B) Molecular Function, C) Cellular Component, D) KEGG pathway.

**Fig. 4:**
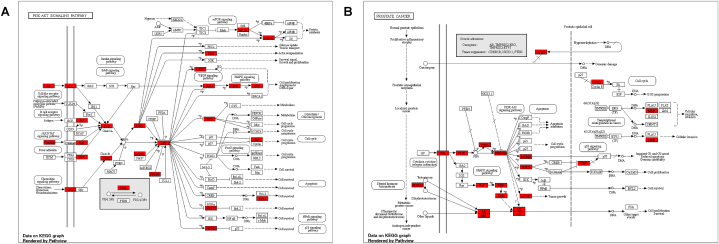
A) PI3K-Akt signaling pathway B) Prostate cancer signaling pathway.

In order to explore the underlying target proteins, GO enrichment analysis was conducted. The target genes were evaluated using three criteria in configuring the Enrichr and ShinyGO settings: GO biological (Table 1 and Fig. 3A), GO molecular (Table 2 and Fig. 3B), and GO cellular (Table 3 and Fig. 3C). Table 4 and Fig. 3D focused on the crucial KEGG pathway. The false discovery rate according to the Benjamini-Hochberg approach directed the fusing of GO keywords to a threshold of p ≤ 0.05. Because of this, we ran extensive GO and KEGG pathways to figure out the signalling pathways, and we found some interesting connections between the PI3K-Akt signalling pathway and prostate cancer (Fig. 4A and B). The polyherbal formulation has shown promise in the treatment of a number of diseases and disorders, such as hepatitis B, bladder cancer, breast cancer, Alzheimer's disease, ovarian steroidogenesis, lipid and atherosclerosis, and human cytomegalovirus infection. The extensive KEGG research showed their participation in numerous disease pathways and diseases, while they were originally chosen to examine impacts linked to prostate cancer. The use of Polyherbal formulation as new pharmacotherapeutic agents shows potential for the treatment of various diseases and disorders, considering these complex connections and the visualised network.

**Table 1:** Go biological process.

Description	P-value
Protein Phosphorylation	8.855 × 10^−41^
Phosphorylation	7.821 × 10^−35^
Protein Modification Process	3.210 × 10^−27^
Regulation Of Apoptotic Process	1.512 × 10^−26^
Positive Regulation Of Intracellular Signal Transduction	1.139 × 10^−24^
Protein Autophosphorylation	1.284 × 10^−24^
Regulation Of Cell Population Proliferation	4.145 × 10^−23^
Negative Regulation Of Programmed Cell Death	7.466 × 10^−23^
Transmembrane Receptor Protein Tyrosine Kinase Signaling Pathway	2.259 × 10^−21^
Negative Regulation Of Apoptotic Process	7.659 × 10^−21^

**Table 2:** Go molecular process.

Description	P-value
Protein Serine/Threonine Kinase Activity	7.129 × 10^−30^
Protein Tyrosine Kinase Activity	3.610 × 10^−20^
Transmembrane Receptor Protein Tyrosine Kinase Activity	8.175 × 10^−15^
Heme Binding	1.252 × 10^−13^
Transition Metal Ion Binding	5.926 × 10^−12^
Estrogen Response Element Binding	1.485 × 10^−11^
Protein Kinase Binding	2.060 × 10^−11^
Endopeptidase Activity	8.550 × 10^−10^
Transmembrane Receptor Protein Kinase Activity	2.248 × 10^−9^
Protein Homodimerization Activity	1.109 × 10^−8^

**Table 3:** Go cellular components.

Description	P-value
Intracellular Membrane-Bounded Organelle	1.139 × 10^−18^
Nucleus	9.325 × 10^−15^
Membrane Raft	4.629 × 10^−10^
Ficolin-1-Rich Granule Lumen	2.454 × 10^−7^
Caveola	2.667 × 10^−7^
Plasma Membrane Raft	3.095 × 10^−7^
Endoplasmic Reticulum Membrane	7.626 × 10^−7^
Endosome Lumen	0.000001264
Secretory Granule Lumen	0.000004221
Focal Adhesion	0.000004365

**Table 4:** KEGG pathways.

Description	P-value
Pathways in cancer	1.363 × 10^−44^
Chemical carcinogenesis	2.125 × 10^−28^
PI3K-Akt signaling pathway	4.674 × 10^−24^
Human cytomegalovirus infection	2.124 × 10^−21^
Lipid and atherosclerosis	6.672 × 10^−21^
Prostate cancer	2.235 × 10^−20^
Proteoglycans in cancer	2.538 × 10^−19^
HIF-1 signaling pathway	3.379 × 10^−19^
Kaposi sarcoma-associated herpesvirus infection	6.043 × 10^−19^
AGE-RAGE signaling pathway in diabetic complications	9.279 × 10^−19^

### 3.4 Molecular docking

**Fig. 5:**
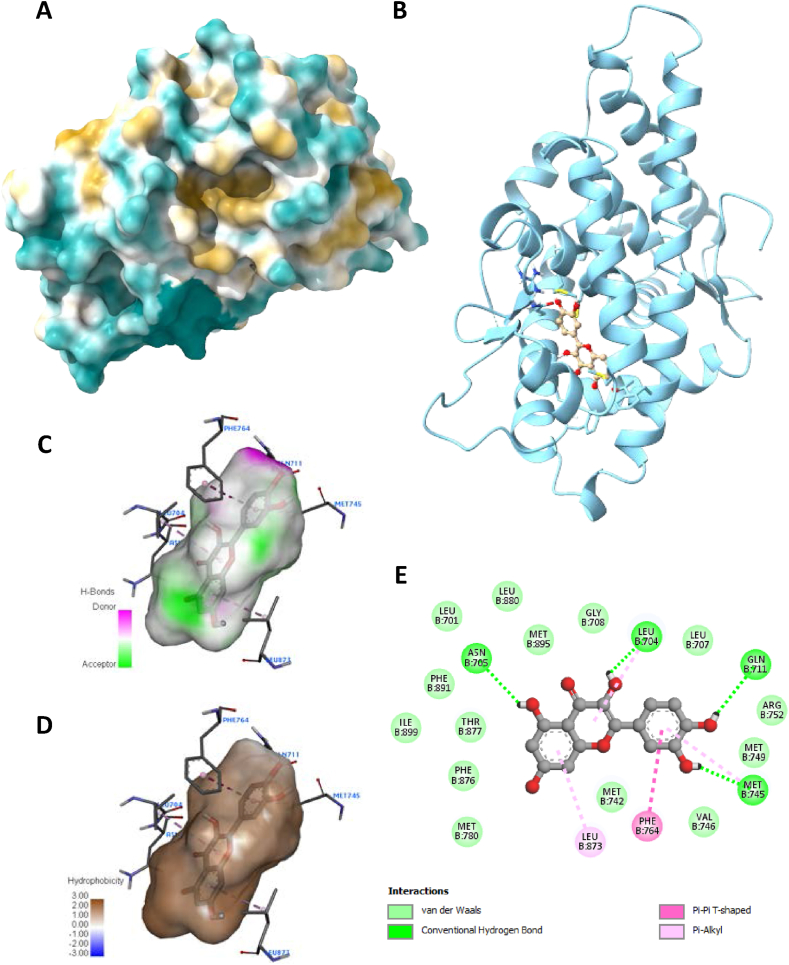
Visualization of docking analysis of Quercitin binding with 5JJM (A) hydrophobicity surface 3D representation (B) interaction of Quercitin with 5JJM (C) visualization of hydrogen bond (D) visualization of hydrophobic interaction (E) 2D representation describing bindings of Quercitin with active site of 5JJM.

**Fig. 6:**
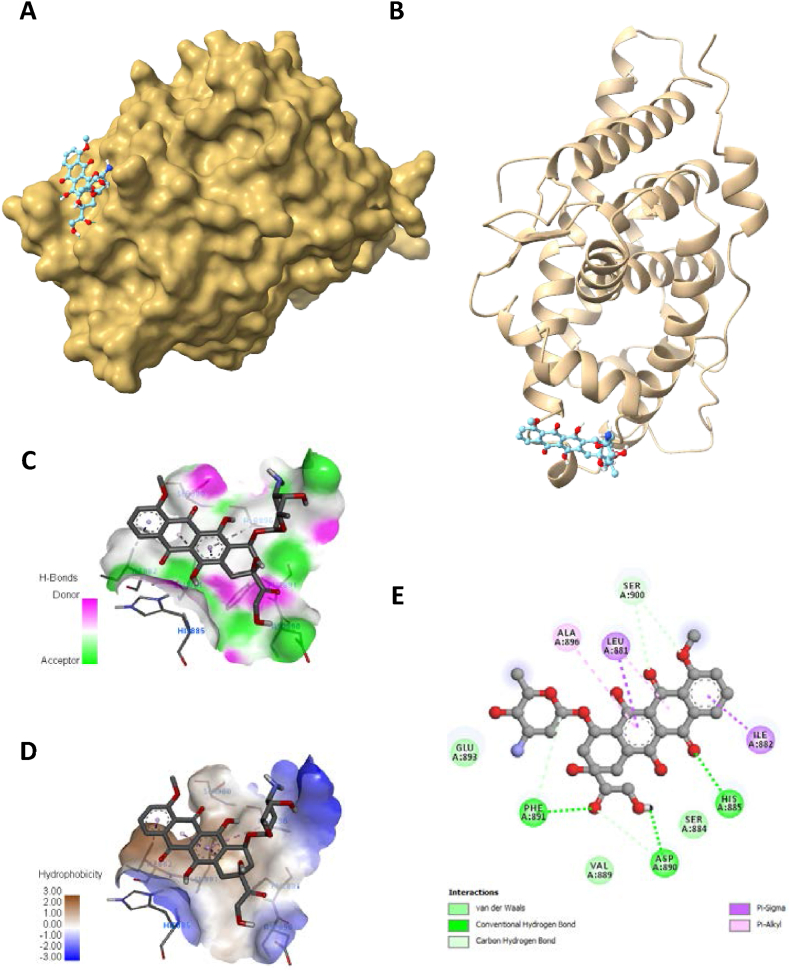
Visualization of docking analysis of Doxorubicin binding with 5JJM (A) hydrophobicity surface 3D representation (B) interaction of Doxorubicin with 5JJM (C) visualization of hydrogen bond (D) visualization of hydrophobic interaction (E) 2D representation describing bindings of Doxorubicin with active site of 5JJM.

**Fig. 7:**
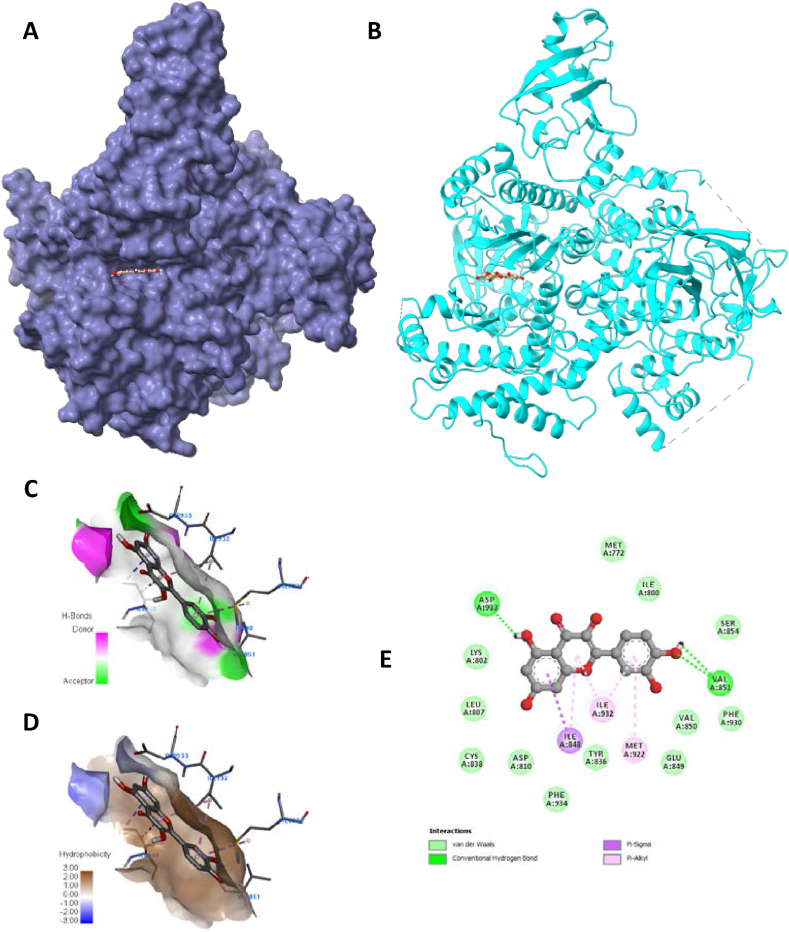
Visualization of docking analysis of Quercitin binding with 4JPS (A) hydrophobicity surface 3D representation (B) interaction of Quercitin with 4JPS (C) visualization of hydrogen bond (D) visualization of hydrophobic interaction (E) 2D representation describing bindings of Quercetin with active site of 4JPS.

**Fig. 8:**
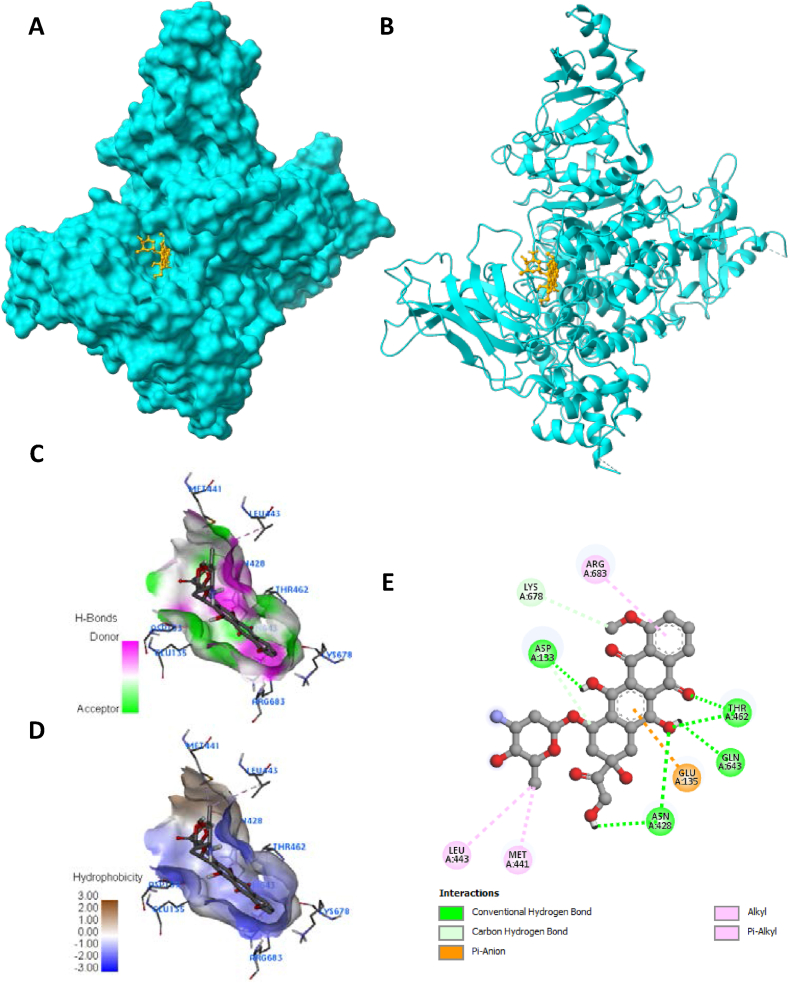
Visualization of docking analysis of Doxorubicin binding with 4JPS (A) hydrophobicity surface 3D representation (B) interaction of Doxorubicin with 4JPS (C) visualization of hydrogen bond (D) visualization of hydrophobic interaction (E) 2D representation describing bindings of Doxorubicin with active site of 4JPS.

It is necessary to investigate the molecular component's structural basis and its ability to predict the binding conformation of low-molecular-weight ligands to their target binding sites after a thorough investigation of the complex signalling pathways and pathological states linked with individual genes. The proteins in question were chosen for their significant contributions to the complex mechanism of prostate cancer, as well as their functions in protein-protein interaction (PPI), the compound-target network, and the KEGG analysis. Active components docking scores and conformations with regard to their interactions with PIK3R1 (PDB: 4JPS) and AR (PDB: 5JJM) are described in detail in (Table 5), with representation in (Fig. 5, Fig. 6, Fig. 7, Fig. 8). These components are Thymoquinone, Nigellicine, Nigellidine, Nigellimine, Catechin, Quercetin, Luteolin, Ajoene, and Allixin.

**Table 5:** Estimated Free Energy of Binding, H-Bond Interactions and Hydrophobic Interactions between compounds and receptors.

Targets	Compounds	Pubchem ID	Binding Affinity	Hydrophobic Interactions	Hydrogen bonding
AR	Ajoene	5386591	−4.2	PRO694,ASN727,PHE826	GLN693,ASP695
	Allixin	86,374	−6.3	LEU704,LEU707,MET749,PHE764,LEU873	GLY708
	Catechin	9064	−8.5	LEU701,MET745,PHE764,LEU873	LEU707,LEU873,THR877
	Luteolin	5,280,445	−8.3	VAL684,VAL715,ARG752	PRO682,GLN711,TRP751,LYS808
	Nigellicine	11,402,337	−7.6	MET745,VAL746,MET749,PHE764,LEU873	ASN705,GLY708
	Nigellidine	136,828,302	−7.5	ALA748,ARG752,LEU805	TRP751,ARG752,ASN756
	Nigellimine	20,725	−5.5	GLU706, ASP890	ASP690, GLU706
	Quercetin	5,280,343	−9	MET745	LEU704,GLY708,GLN711,MET745,ARG752,LEU873
	Thymoquinone	10,281	−6.3	LEU704,LEU707,VAL746,PHE764,LEU873	PHE764
	Doxorubicin	31,703	−7.1	LEU881,ILE882,PHE891,ALA896	HIS885, PHE891
PIK3R1	Ajoene	5386591	−4.7	ILE800,TYR836,ILE848,ILE932,	VAL851, SER854
	Allixin	86,374	−6	LEU704,LEU707,MET749,PHE764,LEU873	GLY708
	Catechin	9064	−8.5	LEU701,MET745,PHE764,LEU873	LEU707,LEU873,THR877
	Luteolin	5,280,445	−8.5	VAL684,VAL715,ARG752	PRO682,GLN711,TRP751,LYS808
	Nigellicine	11,402,337	−7.4	MET745,VAL746,MET749,PHE764,LEU873	ASN705,GLY708
	Nigellidine	136,828,302	−8.1	ALA748,ARG752,LEU805	TRP751,ARG752,ASN756
	Nigellimine	20,725	−6.5	GLU706,ASP890	ASP690, GLU706
	Quercetin	5,280,343	−8.4	MET745	LEU704,GLY708,GLN711,MET745,ARG752
	Thymoquinone	10,281	−6.2	LEU704,LEU707,VAL746,PHE764,LEU873	PHE764
	Doxorubicin	31,703	−9.5	TRP780,ILE800,ILE848,VAL850,ASN920,ILE932,ASP933	SER774,GLU849,VAL851,GLN859,ASP933

### 3.5 Molecular dynamic simulation

**Fig. 9:**
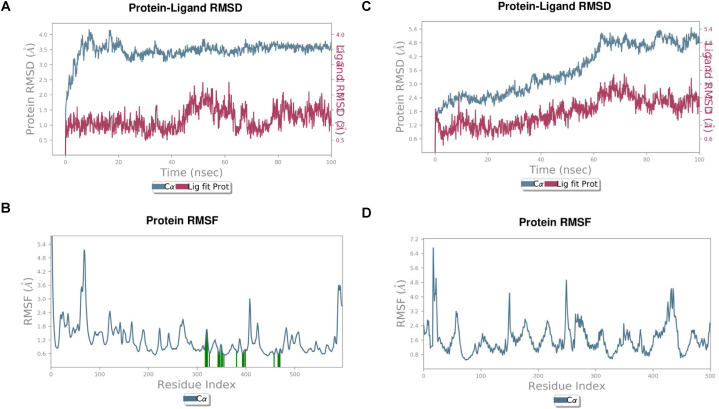
RMSD/RMSF values during molecular dynamics simulation (A,B) Quercetin-4JPS complex, (C,D) Quercetin-5JJM complex.

We chose the Androgen receptor (AR: PDB-5JJM) and phosphoinositide-3-kinase regulatory subunit 1 (PIK3R1: PDB-4JPS) for molecular dynamics simulation to further study the interaction between the active compound Quercetin and the target protein. This decision was based on the results of network pharmacology and molecular docking. Using molecular dynamics simulation, we compared and evaluated the complexes and unoccupied protein's stability, structural changes, and residual fluctuations across a 100 ns simulation. We examined the trajectories of the simulated complexes using various standard simulation parameters, including root-mean square fluctuations (RMSFs), intermolecular interactions, protein-ligand contacts, solvent accessible surface area (SASA), and radius of gyration (rGyr). We examined the simulation data for trajectories. On the coupled Quercetin-4JPS complex, a molecular dynamics simulation was run for 100 nanoseconds. The analysis showed that when the ligand was bound, the Cα atoms of the protein's Root Mean Square Deviation (RMSD) stabilised with only small changes (Fig. 9A). After 45–62 ns, the ligand RMSD fluctuated somewhat between 0.5 and 2.3 Å, before stabilising at 100 ns and displaying an average RMSD between 0.5 and 1.7 Å. As shown in (Fig. 9B)the RMSF value of the Quercetin-4JPS complex was computed. According to MD trajectories, the amino acid residues in the 50 to 60 region exhibit variations, whereas the higher peaks of the residues in the loop sections, N- and C-terminal zones indicate stability. Low RMSF values, averaging between 0.6 and 2.4 Å, indicated that the chosen ligand binding stability against the target protein was satisfactory. In contrast, the Quercetin-5JJM complex RMSD (Fig. 9C) first remains stable for 62 ns with an RMSD of 0.6–2.4 Å, then exhibiting small variations for 75 ns before stabilising for 100 ns.Also computed from the trajectories was the RMSF of the Quercetin-5JJM complex (Fig. 9D), with fluctuations seen in the amino acid residues located in the 150, 250, and 440 region. Low RMSF values, averaging between 0.8 and 3.2 Å, indicated that the chosen ligand binding stability against the target protein was satisfactory.

The docked pose of the Quercetin-4JPS complex showed strong hydrogen bonds with ASP810 (89 %), VAL851 (70 %), GLU849 (32 %), LYS802 (26 %), and TYR836 (23 %), according to the 2D ligand interaction diagram (Fig. S1A) of the 100ns simulation. (Fig. S1B) shows that there are substantial hydrogen bonds between Quercetin-5JJM and ASN705 (87 % and 78 %, respectively). Furthermore, along the stimulation trajectory of TRP780, ILE800, LUE807, TYR836, ILE848, VAL850, MET922, PHE930, and ILE932, there were hydrophobic bonds for the Quercetin-4JPS complex. The ligand protein contacts MET722, SER774, LYS776, LYS802, ASP805, ASP806, ASP810, GLU849, VAL851, SER854, and ASP933 formed extensive water bridge interactions. Hydrophobic bindings were found between several Quercetin-5JJM complex components, including LEU701, LEU704, LEU707, MET742, MET745, PHE764, MET780, LEU873, PHE876 and LEU880. The clonazepam protein contacts included GLN711, MET745, ARG752, PHE764, SER778 and PHE876. Water bridge interactions were also abundant. These hydrogen bonds are crucial for stabilising Quercetin in the protein pocket, as shown by the strong interactions between Quercetin and ASN705, LYS802, ASP810, VAL851, and THR877 in the ligand interaction fraction (Figs. S2A–B). The complex may be stabilised by the strong hydrophobic contacts that Quercetin displayed with LEU701, LEU704, MET742, PHE764, TYR780, GLU798, ILE848, MET922, and ILE932.

(Figs. S3A–B) shows the amounts of time that each amino acid residue interacts with the Quercetin-4JPS and Quercetin-5JJM complex. Evidence of the binding between the selected protein and ligand was provided by the molecular associations and linkages, which included hydrophobic interactions, water bridges, ionic interactions, and hydrogen bonds. The interactions between the ligands were revealed by computing the location of each photo along the x-axis. On top of that, we found a number of different interactions with the ligand. The simulation for both Complex was conducted with constant monitoring of the secondary structure elements (SSEs), including beta strands and alpha helices. Referring to (Figs. S4A–B), the graphic depicts the distribution of SSEs throughout the protein structure as a function of residue index. In this form, the SSE composition is captured for each simulation trajectory frame, and in the plot that follows, the SSE assignment for each residue is tracked over time. Alpha helices made up 39.12 % of the Quercetin-4JPS complex, beta sheets 7.34 %, and miscellaneous secondary structural components 46.46 %. In contrast, of the Quercetin-5JJM complex, 56.55 % was secondary structure, 2.28 % was beta sheets, and 54.27 % was alpha helices. One important factor influencing the protein's Root Mean Square Deviation (RMSD) is the ratio of alpha helices to beta sheets. In addition, Fig. S5 A-B provides a detailed representation of the ligand characteristics. See Table 6 for more information on how the MM-GBSA approach was used to obtain the complexes' minimized binding free energy.

**Table 6:** MM–GBSA.

Complex	Time (ns)	MMGBSA-dG-binding energy	MMGBSA-dG-bind in Coulomb	MMGBSA-dG-bind(NS)	MMGBSA-dG bind(NS)-Coulomb
Quercetin-4JPS	0	−67.58368523	−30.40368924	−72.79682512	−31.16268901
Quercetin-4JPS	100	−64.56155572	−19.40946215	−67.71882363	−21.23751906
Quercetin-5JJM	0	−87.58574964	−22.21918564	−92.04201574	−23.57556717
Quercetin-5JJM	100	−86.48537098	−19.75124784	−91.29387019	−22.37822054

The relative binding-free energies (kcal/mol) obtained by MM–GBSA, where MMGBSA dG Bind = Complex – Receptor – Ligand and MMGBSA dG Bind (NS) = Complex – Receptor (from optimized complex) – Ligand (from optimized complex) = MMGBSA dG Bind − Receptor Strain − Ligand Strain. NS in the table is no strain; it is the binding energy without considering for the receptor and ligand conformational changes needed for the formation of complex.

## 4 Discussion

As a common cancer with potentially fatal effects, prostate cancer is a major concern for men's health [22]. It has the second-highest incidence rate of any cancer among men and is thus the most common [23]. It is of the utmost importance to improve treatment outcomes and decrease mortality rates for this illness. Network pharmacology (NP) is an alternative to the traditional “one drug, one target" approach to drug design that aims to investigate the complex interplay between medications and diseases through the use of multi-targeted treatment. This fresh method combines redundancy, connection, systems biology, and network analysis. New targets and compound-associated signalling pathways have been successfully uncovered by NP. It provides new insights into the interconnected systems of therapeutic targets and diseases, making it a powerful tool for unraveling the underlying mechanisms of diseases and finding new bioactive compounds. Consistent with this, our research provides a holistic perspective by presenting a novel network that describes the molecular process of a Polyherbal Formulation.

Among the polyherbal ingredients are garlic, honey, and Nigella Sativa. The active components derived from these substances were tested according to DL and OB standards. Nine active components were found to be suitable for further investigation: Ajoene, Allixin, Catechin, Luteolin, Nigellicine, Nigellidine, Nigellimine, Quercetin, and Thymoquinone. A prostate cancer-related network was built using plant bioactive targets; this network reveals that nine polyherbal bioactives have the ability to affect prostate cancer through several pathways involving 845 proteins. In vitro studies with different cancer cell types have shown that these components can suppress cell proliferation, induce cell cycle arrest, and promote apoptosis. An investigation of protein-protein interactions (PPI) was used to identify 291 nodes and 616 edges. Some of the genes that have been linked to the development of prostate cancer are PIK3R1, SRC, STAT3, HSP90AA1, AKT1, MAPK1, SESR1, and AR. Furthermore, a plethora of pathways and diseases/disorders linked to the chosen genes were revealed by GO and KEGG studies.

Bioactives play a direct role in prostate cancer regulation, according to the GO enrichment analysis. According to the KEGG pathway analysis, the selected network may include the PI3K-Akt signalling route and the Prostate cancer signalling pathway, both of which lend credence to the idea that the polyherbal formulation could be useful in the treatment of prostate cancer. Proliferation, metastasis, inflammation, and angiogenesis are all biological processes that can be accelerated by the aberrant activation of these targets, which in turn can worsen the malignant process of prostate cancer. The various signalling pathways that were identified include: PI3K-Akt, Prostate cancer, Ras, Chemokine, HIF-1, FoxO, sphingolipid, AMPK, VEFG, JAK-STAT, insulin, GnRH, oestrogen, prolactin, thyroid hormone, relaxin, progesterone-mediated oocyte maturation, and progesterone. This suggests that the polyherbal formulation may be used for multi-targeting.Cancers of the colorectal, renal, pancreatic, endometrial, melanoma, bladder, small cell, non-small cell, hepatocellular, and gastric varieties are all possible with the use of polyherbal formulation. According to [24], androgens play a role in the development of prostate cancer, a malignant tumor. This cancer's growth and metastasis are driven in large part by the androgen receptor (AR) [25]. The process of prostate cancer cell proliferation, differentiation, and metastasis is aided by androgen-mediated transcription of AR target genes [26]. For this reason, AR therapy is an essential tool in the fight against prostate cancer.

Furthermore, targets were validated using the docking investigation. Additionally, it checks for component-target affinity, which can shed light on the structure-activity link. Androgen receptor (AR: PDB-5JJM) and phosphoinositide-3-kinase regulatory subunit 1 (PIK3R1: PDB-4JPS) docked ligands have free energies of binding ranging from −4.2 to −9 kcal/mol and −4.7 to −9.5 kcal/mol, respectively, according to the molecular docking analysis. Hydrophobic interactions, hydrogen bond formation, and other important interactions, such as π-stacking and salt bridge building, were implicated in the observed binding patterns between the bioactive chemicals and the target proteins. It is worth noting that our data particularly within the experimental series, quercetin showed the strongest affinity for all of the receptors indicated above. Quercetin interacts with a larger number of important amino acid residues, which is why it has a higher binding affinity. Significantly, it was determined that a higher inhibitory potential was associated with a higher number of hydrogen bonds. A closer look at the docking scores revealed that Quercetin had a significantly lower binding energy of −9 kcal/mol to the Androgen receptor than the standard doxorubicin (−7.1 kcal/mol) and a significantly lower binding energy of −8.5 kcal/mol to phosphoinositide-3-kinase regulatory subunit 1 than the standard drug doxorubicin (−9.5 kcal/mol). There were six major hydrogen bonds formed by the quercetin-5JJM complex with important amino acid residues, including LEU704, GLY708, GLN711, MET745, ARG752, and LEU873 (Fig. 5A–E), in contrast to the two hydrogen bonds shown by the conventional compound doxorubicin (Fig. 6A–E). The Quercetin-4JPS complex interaction was aided by the formation of five pivotal hydrogen bonds with essential interacting amino acid residues, specifically LEU704, GLY708, GLN711, MET745 and ARG752 (Fig. 7A–E). While the standard compound doxorubicin exhibited five hydrogen bonds—SER774, GLU849, VAL851, GLN859, and ASP933 (Fig. 8A–E).

The specificity of drug candidates with the residues of the target protein's active site is greatly influenced by the establishment of hydrogen bonds and hydrophobic interactions. A ligand-protein complex's binding affinity and stability are enhanced by hydrogen bonds, which allow for targeted interactions between the ligand and the active site's amino acid residues. The binding specificity is further improved by hydrophobic interactions between the ligand's nonpolar domains and the protein, which optimise the complementarity between the two and the active site. To design powerful and selective drug candidates in molecular docking studies, it is crucial to understand and optimise these interactions. For compound Quercetin-5JJM, the binding residues were determined to be ASN705, GLN711, MET745 and THR877, while for complex Quercetin-4JPS, the essential binding residues were LYS802, ASP810, TYR836, GLU849, VAL851, SER854, ASP933 and PHE934. The molecule's departure from the active site was illuminated by calculating the root mean square fluctuation (RMSF) and root mean square deviation (RMSD) values. Results showing decreased RMSD and RMSF values across the whole 100 ns MD simulation were indicative of stable drug-ligand complexes and the absence of substantial molecular fluctuations around the protein active site. There were very few variations throughout the whole 100 ns of the MD simulations, proving that the docked complex interactions were stable. In addition, the binding free energy of the Quercetin-4JPS and Quercetin-5JJM complex was meticulously determined using MM/GBSA in order to obtain insights into the interactions between the receptors and inhibitors, specifically the Androgen receptor and PIK3R1. As can be seen in Table 6, the MM-GBSA approach yielded binding free energy data.

The study found that out of all the receptors in the series that were tested, Quercetin had the strongest affinity. An increased inhibitory potential is directly proportional to the amount of hydrogen bonds that are identical. One common method in computational drug design is the use of MD simulations, which allow one to determine the kinetic and thermodynamic properties of biological systems under precise physiological conditions. Molecular dynamics simulation studies were conducted on the top compounds to evaluate the stability of the drug-ligand docked complex. The simulated complexes were consistently accurate, especially at the target protein's binding region. The interaction residues between the receptor protein and the chosen ligands were revealed by molecular docking and MD simulation studies.

## 5 Conclusion

To identify the main target proteins linked to a polyherbal formulation for prostate cancer treatment, we used a thorough methodology that combined network pharmacology with vast database mining tools in this study. The method relied on painstakingly building a network out of target proteins. Among other important biological processes, our findings demonstrated that the polyherbal formulation played a pivotal role in the PI3K-Akt signalling cascade and pathways associated with prostate cancer. These results showed that the polyherbal formulation modulated important target proteins including PIK3R1, AKT1, ESR1, and AR, suggesting that it has great therapeutic promise in the fight against prostate cancer. Also, we looked at the complex process by which the polyherbal formulation controls prostate cancer by influencing many signalling pathways and targeting numerous proteins. This data points to the polyherbal formulation's promise as an innovative anti-tumor agent for prostate cancer treatments. The polyherbal formulation shows potential as a new pharmacological intervention in the treatment of prostate cancer, according to the combined findings of dynamic simulation studies, molecular docking, and network analysis. We need more studies to fill in the gaps in our knowledge and find better ways to treat prostate cancer.

## 6 Limitations and future work

Our paper presents a thorough computational methodology for the identification of compounds with the potential to treat prostate cancer. We also provide a predictive mechanism of action for these compounds. Several limitations of our study are something we are fully aware of. We have tried our best to construct comprehensive drug-target networks from publicly available sources, however it is still possible that some drug-target interactions that are biologically relevant may have been overlooked due to inadequate network data. The suggested network-based approaches would be far more accurate with more precise cancer cell identification and their unique biological impacts measured by functional genomics assays. To determine the in vivo effectiveness and possible adverse effects by means of cell validation tests, preclinical research is necessary prior to clinical trials. Therefore, in order to support the idea in future study, it is crucial to conduct additional experimental validation of network pharmacology predictions.

## Ethics approval and consent to participate

Not applicable.

## Consent for publication

Not applicable.

## Funding statement

The authors have no relevant financial or nonfinancial interests to disclose.

## Data availability

All the data generated or analyzed during this study are included in this article.

## CRediT authorship contribution statement

**Ansari Vikhar Danish Ahmad:** Writing – review & editing, Writing – original draft, Software, Investigation, Data curation, Conceptualization. **Syed Ayaz Ali:** Writing – review & editing, Supervision, Investigation. **Qazi Yasar:** Visualization, Software. **Nikhil S. Sakle:** Visualization, Methodology. **Mohd Mukhtar Khan:** Formal analysis.

## Declaration of competing interest

The authors declare that they have no known competing financial interests or personal relationships that could have appeared to influence the work reported in this paper.

## **Abbreviations**

PPI: protein–protein interaction
PDB: Protein Data Bank
MD: Molecular Dynamic
PCa: Prostate cancer
AR: Androgen Receptor
CRPC: Castration-resistant prostate cancer
OB: Oral bioavailability
DL: Drug Likeness
GO: Gene ontology
KEGG: Kyoto Encyclopedia of Genes and Genomic analysis
TIP3P: Intermolecular Interaction Potential 3 Points Transferable
ps: picoseconds
RMSD: Root Mean Square Deviation
MM-GBSA: molecular mechanics generalized Born surface area
SRB: Sulforhodamine B
PHF: Polyherbal Formulation
