# Supplementary material for: Integrative network pharmacology, molecular docking, and dynamic simulation analysis of a polyherbal formulation for potential therapeutic impact on prostate cancer
Source: Heliyon. 2024 Jul 11;10(14):e34531. doi: 10.1016/j.heliyon.2024.e34531 (PMC11305312; doi:10.1016/j.heliyon.2024.e34531)
Supplement: Multimedia component 1 [file mmc1.pptx]

## Slide 1
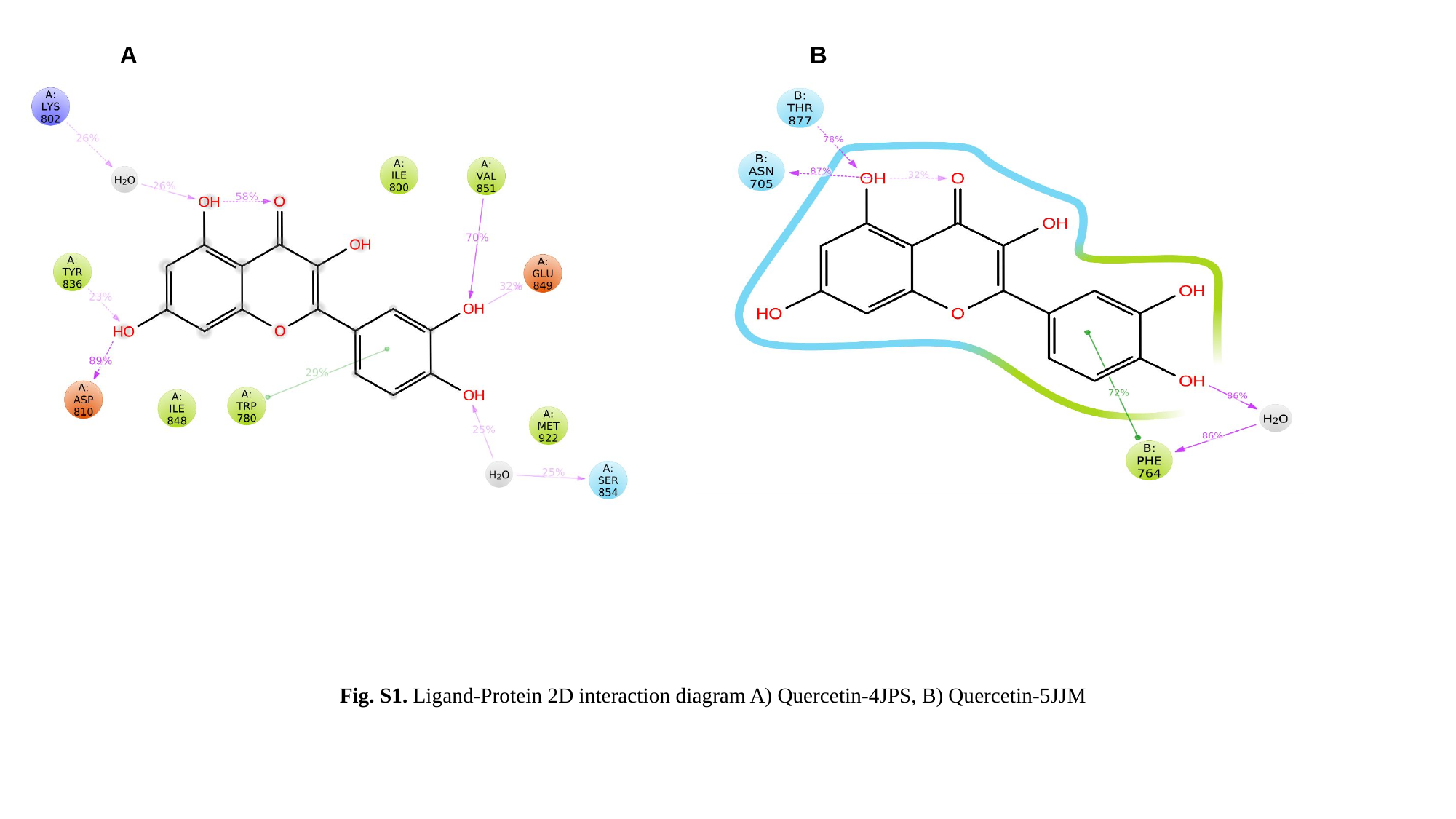

A
B
Fig. S1. Ligand-Protein 2D interaction diagram A) Quercetin-4JPS, B) Quercetin-5JJM

## Slide 2
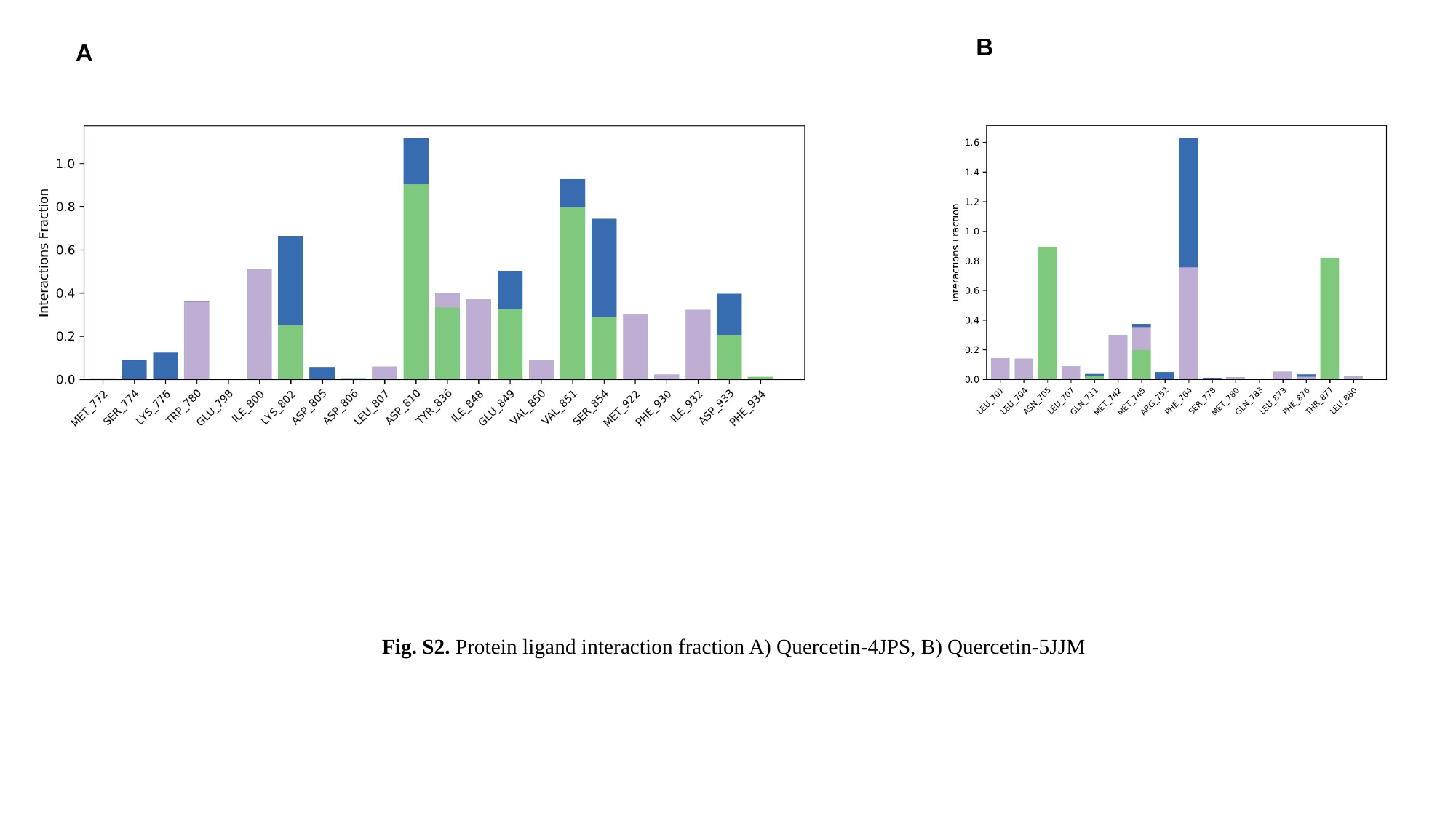

B
A
Fig. S2. Protein ligand interaction fraction A) Quercetin-4JPS, B) Quercetin-5JJM

## Slide 3
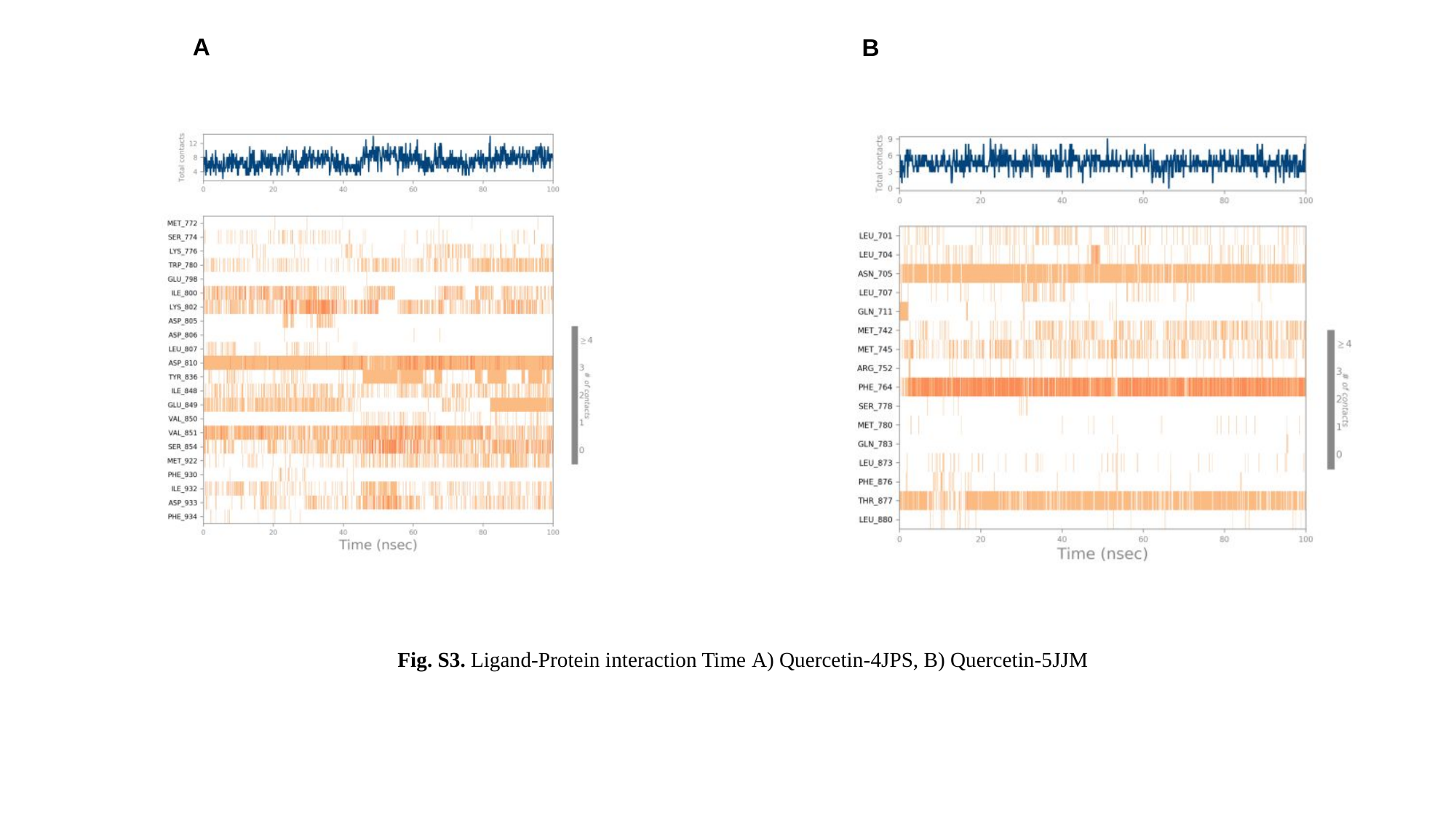

A
B
Fig. S3. Ligand-Protein interaction Time A) Quercetin-4JPS, B) Quercetin-5JJM

## Slide 4
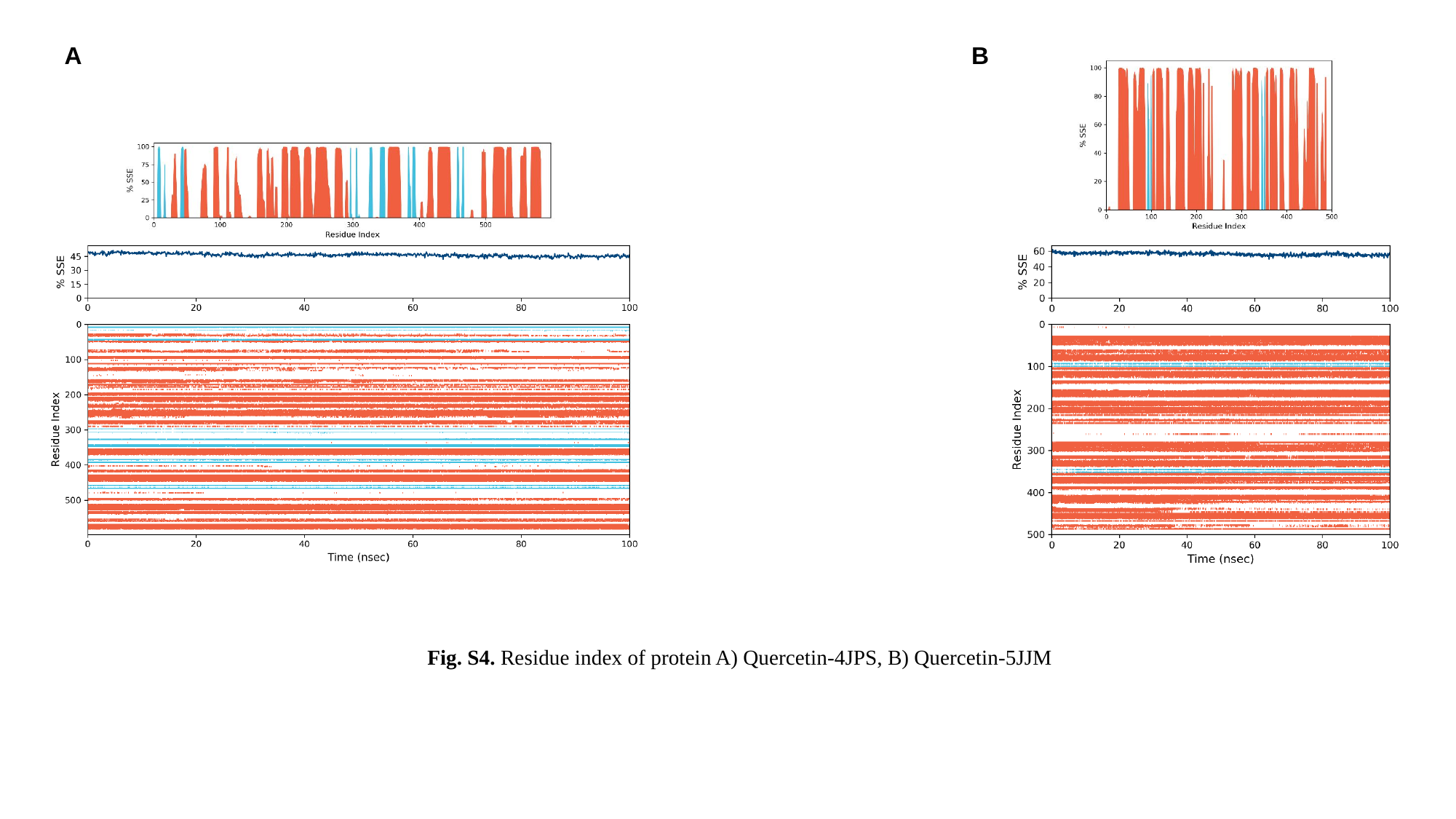

A
B
Fig. S4. Residue index of protein A) Quercetin-4JPS, B) Quercetin-5JJM

## Slide 5
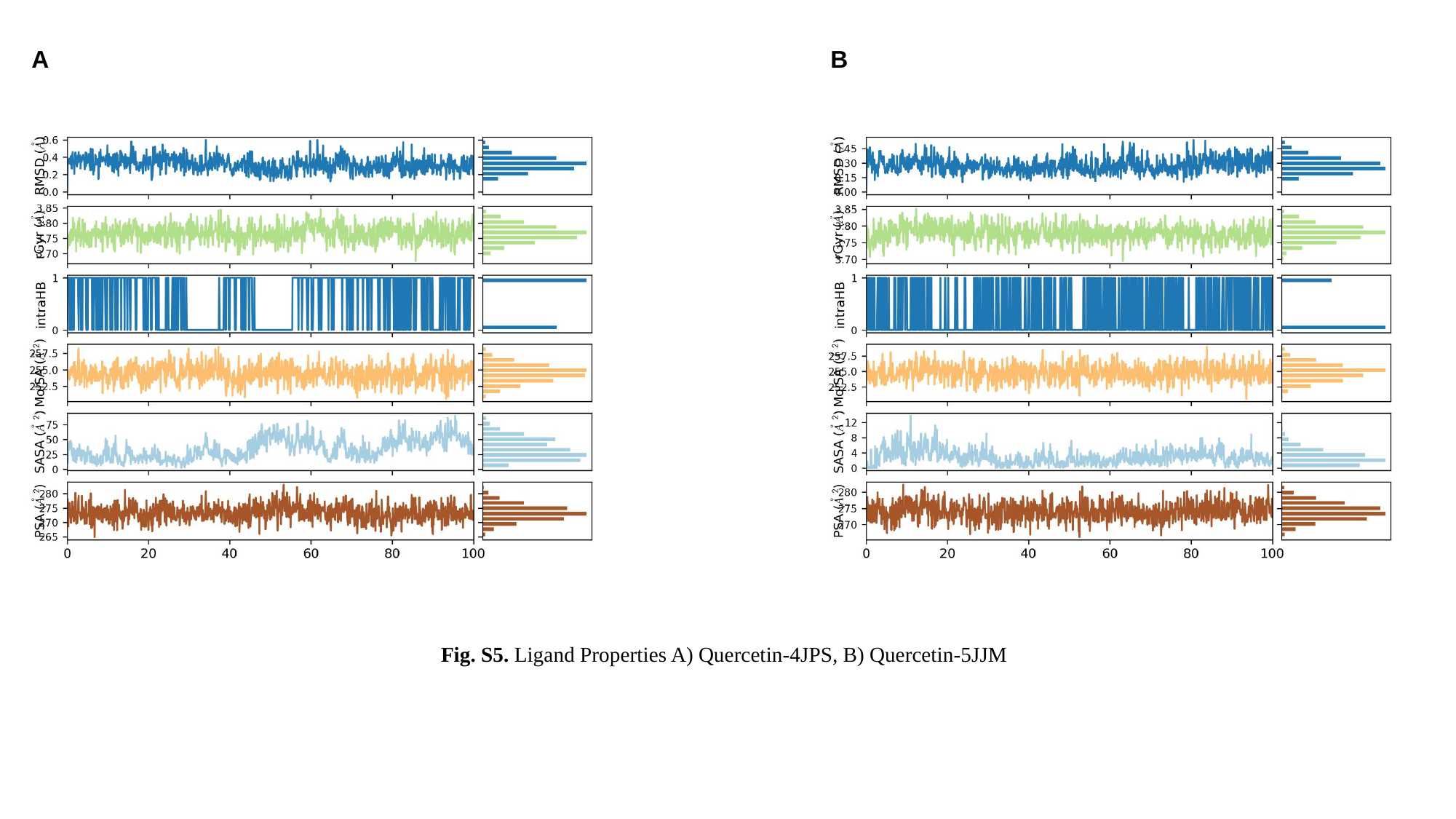

B
A
Fig. S5. Ligand Properties A) Quercetin-4JPS, B) Quercetin-5JJM
